# Composite Lymphoma with Follicular Lymphoma Transformation to Clonally Related Epstein–Barr Virus (EBV) Positive Diffuse Large B-Cell Lymphoma and EBV-PositiveClassic Hodgkin Lymphoma

**DOI:** 10.1155/2023/8833273

**Published:** 2023-11-08

**Authors:** Christopher B. Ryder, Hayder Saeed, Mohammad Hussaini

**Affiliations:** H. Lee Moffitt Cancer Center, 12902 Magnolia Drive, Tampa, FL 33612, USA

## Abstract

While the Epstein–Barr virus (EBV) is known to drive *de novo* lymphomagenesis, it may rarely contribute to transformation of indolent lymphoma as well. Some EBV-related lymphomas represent a diagnostic challenge with important prognostic and therapeutic implications. We describe a case of follicular lymphoma (FL) transformation to both EBV + diffuse large B-cell lymphoma (DLBCL) and EBV + classic Hodgkin lymphoma (cHL), the latter of which was only identified retrospectively after selective outgrowth during DLBCL therapy. Finally, we describe successful salvage therapy with brentuximab vedotin plus nivolumab. This is the first known case of composite lymphoma with FL, EBV + DLBCL, and EBV + cHL within a single lymph node. The disease course highlights the importance of careful morphologic examination and comprehensive immunophenotypic characterization of EBV + lymphomas to ensure proper clinical care and underscores the potential for novel therapies currently under investigation. This trial is registered with NCT01671813.

## 1. Introduction

Epstein–Barr virus (EBV) exerts a well-established role in the *de novo* pathogenesis of various aggressive lymphomas [1]. However, its function in the transformation of indolent lymphomas is less appreciated. Definitive classification of EBV + lymphomas can be challenging, with disparate treatments being indicated for some differential diagnostic considerations. Here, we describe the first known case of a composite lymphoma with both EBV + diffuse large B-cell lymphoma (DLBCL) and EBV + classic Hodgkin lymphoma (cHL) arising in a background of follicular lymphoma (FL), with the cHL component only identified retrospectively following pure EBV + cHL relapse. Furthermore, we demonstrate a clonal relationship between all three lymphoma morphologies and report successful salvage therapy of relapsed cHL with brentuximab vedotin plus nivolumab.

## 2. Case Presentation

The patient is a 54-year-old male who presented to an outside institution with pruritus, fatigue, and cervical lymphadenopathy. On labs, he was found to have anemia, renal insufficiency, and hypercalcemia. Excisional biopsy of the left cervical lymph node identified grade 3A follicular lymphoma (FL) in a follicular pattern. However, a staging positron emission tomography (PET) scan identified multifocal hypermetabolic lymphadenopathy above and below the diaphragm without mediastinal involvement measuring up to 6.7 cm by 5 cm and with a max intensity of 20.2 SUV, as well as mixed sclerotic and lytic bony lesions with SUV up to 26.8. Due to concern for aggressive disease transformation, an excisional biopsy of a hypermetabolic right inguinal lymph node was performed prior to initiation of therapy.

**Figure 1:**
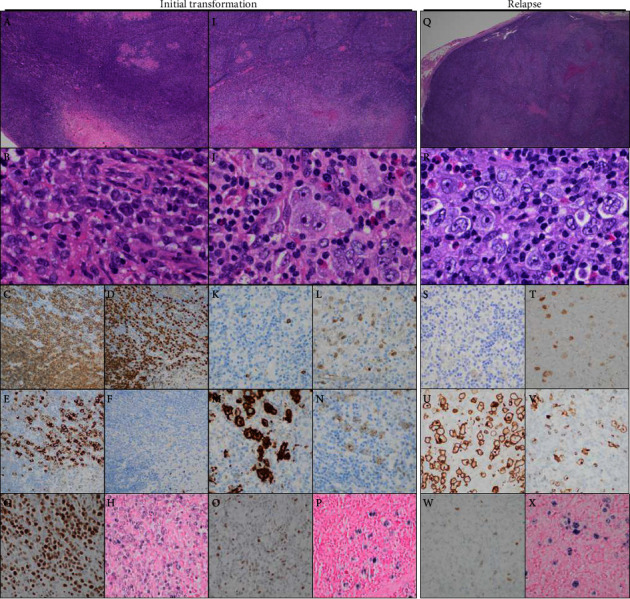
Histology and immunophenotype of initial transformation to EBV + DLBCL (A–H) and EBV + cHL (I–P), as well as relapsed EBV + cHL (Q–X). H & E-stained images at 20x (A, I, Q) and 400x (B, J, R) magnification. Immunostains for CD20 (C, K, S), PAX-5 (D, L, T), CD30 (E, M, U), CD15 (F, N, V), and OCT-2 (G, O, W) and in situ hybridization for EBER (H, P, X), all at 200x.

The biopsied right inguinal node once again showed areas of grade 3A FL (top parts of Figure 1(A, I)), and flow cytometry detected a clonal B-cell population expressing CD10, CD20, and a lambda light chain. In addition to FL, a region around the periphery of the node, comprising 5–10% of the tissue, showed diffuse architecture with central necrosis. Part of this diffuse proliferation was composed of sheets of large lymphoid cells with vesicular chromatin and variably prominent nucleoli (Figure 1(A, B)). These cells were positive for CD20 (Figure 1(C)), PAX-5 (Figure 1(D)), CD30 (Figure 1(E)), OCT-2 (Figure 1(G)), and EBER (Figure 1(H)) and negative for CD15 (Figure 1(F)). The large lymphoma cells were also positive for BCL-6 (weak), BCL-2, and MUM1 and negative for CD10 (not shown). These findings were consistent with a composite lymphoma suggestive of FL transformation to EBV + DLBCL. Areas with FL only were negative for EBER but showed scattered CD30 positive cells within the neoplastic follicles. Outside FISH studies were positive for BCL6 gene rearrangement (80%) and a subclonal gain of BCL2 (20%) and negative for BCL2 and MYC rearrangements.

**Figure 2:**
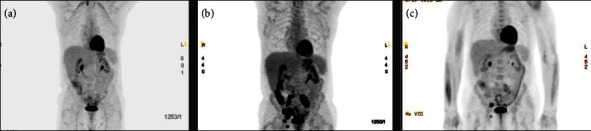
Representative PET reconstructions showing partial response after 6 cycles of R-CHOP (a), disease persistence following second line therapy (b), and complete metabolic response after brentuximab-nivolumab (c).

Following the diagnosis of EBV + DLBCL, the patient was treated at the outside institution with rituximab, cyclophosphamide, doxorubicin, vincristine, and prednisone (R–CHOP) [2]. Interval PET scans revealed partial metabolic response after 2 cycles, mixed response with possible radiologic progression in the right iliac after 4 cycles, and disease progression after 6 cycles (Figure 2(a)). The patient was subsequently enrolled on a randomized phase 3 clinical trial comparing the CD19-targeted antibody-drug conjugate loncastuximab plus rituximab versus rituximab, gemcitabine, and oxaliplatin. By PET, the patient showed a mild improvement after 4 cycles and disease persistence after 5 cycles (Figure 2(b)). At that time, the patient discontinued treatment on the trial less than one year from initial diagnosis.

The patient subsequently established care at our institution and underwent a right inguinal lymph node excision. Sections showed an enlarged lymph node with architectural effacement by an atypical, vaguely nodular, mixed infiltrate composed of moderately numerous Reed–Sternberg (R-S) cells and variants with background small lymphocytes and eosinophils (Figure 1(Q, R)). Variably dense bands of fibrosis and focal necrosis were present. The lymphoma cells were positive for PAX-5 (weak; Figure 1(T)), CD30 (Figure 1(U)), CD15 (Figure 1(V)), MUM1, BCL-6 (weak), BCL-2, and EBER (Figure 1(X)) and essentially negative for CD19, CD20 (Figure 1(S)), BOB1, CD45/LCA, OCT-2 (Figure 1(W)), CD3, and CD10. The morphologic and immunophenotypic findings were consistent with a syncytial variant of EBV + cHL. There was no evidence of FL or DLBCL in this specimen by morphology or flow cytometry. The patient's plasma was positive for EBV at 25,029 IU/ml.

Due to the lack of response to therapy and the discrepant morphology between the diagnostic and persistence/relapse specimens, we performed a careful retrospective examination of the initial EBV + DLBCL transformation. In addition to regions demonstrating monomorphic DLBCL, we identified a small portion of transformed lymphoma with distinct morphology, background cellularity, and immunophenotype (Figure 1(I, J)). This area showed scattered R-S cells and variants in a background of small lymphocytes and scattered eosinophils. The lymphoma cells were negative for CD20 (Figure 1(K)), OCT-2 (Figure 1(O)), BOB1, and CD79a and positive for PAX-5 (weak; Figure 1(L)), CD30 (Figure 1(M)), CD15 (Figure 1(N)), MUM1, and EBER (Figure 1(P)). The overall morphology and phenotype of this region were consistent with occult EBV + cHL comprising less than 5% of the involved tissue, resulting in a revised diagnosis of composite lymphoma with both EBV + DLBCL and EBV + cHL arising in a background of FL.

This unusual transformation event was suggestive of divergent clonal evolution of FL, but FISH performed on the relapse specimen was negative for the BCL6 gene rearrangement and gain of BCL2. To further evaluate the clonal relatedness between the patient's historical FL, EBV + DLBCL, and EBV + cHL, B-cell receptor gene rearrangement studies were performed to assess for shared clonal peaks. Extracted DNA from the initial FL diagnostic specimen, macrodissected monomorphic EBV + DLBCL, and relapsed EBV + cHL were analyzed for clonal B-cell gene rearrangements using Biomed II primers. All three specimens shared peaks in both ΚA and ΚB tubes (x2) and, in addition, EBV + DLBCL and EBV + cHL shared peaks for IgH FR1 and FR2 (Table 1). No clonal peaks were identified in the FR1 or FR2 reactions for the initial FL specimen. These data confirmed a clonal relationship between all three lymphoma morphologies.

In light of the new diagnosis of EBV + cHL and the fact that—despite its occult nature on previous biopsy—this disease component proved refractory to treatment with anthracycline-based therapy, the patient was started on brentuximab vedotin plus nivolumab [3] with a complete metabolic response after 2 cycles seen on the PET scan (Figure 2(c)). The patient was subsequently referred for autologous hematopoietic stem cell transplant consolidation.

## 3. Discussion

Composite lymphoma, the coexistence of two or more distinct lymphoma subtypes in a single biopsy, is an uncommon finding. The distinct components of composite lymphomas can include a wide spectrum of lymphoma entities which may or may not be clonally related on a case-by-case basis. While cHL has been reported as a composite lymphoma with either DLBCL or FL, occasionally in association with EBV [4, 5], we believe that this case is the first report of all three lymphomas arising in a single lymph node. Furthermore, we demonstrate a clonal relationship between the three lymphomas using B-cell receptor gene rearrangement studies. The combination of shared clonality and the presence of EBV only in the DLBCL and cHL components support a potential role of EBV in FL transformation in this instance. Finally, while the initial therapy was directed to B-cell lymphoma, R-CHOP contains doxorubicin which is essential for the treatment of Hodgkin lymphoma [6]. The patient's refractoriness to frontline chemotherapy led us to believe that subsequent high-dose chemotherapy unlikely to cure the disease, and thus, a choice of brentuximab vedotin and nivolumab was made [3, 7], which showed initial efficacy as salvage therapy.

EBV exhibits a known association with lymphoma, especially large B-cell lymphomas, cHL and T/NK cell lymphoproliferative disorders [1]. A pathogenic role has been established in lymphomagenesis, particularly in the setting of compromised immunity. Case reports suggest that the virus may occasionally contribute to aggressive disease transformation, not only to *de novo* pathogenesis [8–10]. In the case presented herein, we hypothesize that the immunologically tolerogenic microenvironment of FL may have enabled EBV reactivation, as the patient had no other identified immunosuppressive state, nor had he begun chemotherapy at the time of composite lymphoma diagnosis. Previous studies have shown that EBV + DLBCL may rarely ensue from an antecedent FL with a variable number of EBV + cells [9, 10]. In our case, no EBER staining was identified in the initial FL. However, neoplastic follicles did show scattered CD30 positive cells, though the exact identity of the CD30+ cells within neoplastic follicles is uncertain. Interestingly, CD30 positivity has been associated with EBV positivity in FL [10]. Transformation of FL to cHL has also been reported, often with a clonal relationship [11], and EBV may play a role in an FL-to-cHL transition in rare instances [9].

Although B-cell clonality studies showed clonal relatedness among all components of this composite lymphoma, the cHL relapse did not harbor the BCL6 gene rearrangement or gain of BCL2 as identified in the composite lymphoma. This finding suggests that EBV may have supplied different oncogenic factors to drive cHL transformation. While the overall morphologic and genetic findings supported EBV-mediated transformation of FL to both DLBCL and cHL, an alternative hypothesis is that EBV reactivation in a common clonally rearranged lymphoma precursor triggered divergent lymphomagenesis. It remains uncertain whether the BCL6 rearrangement and BCL2 gain were present in FL, DLBCL, or both, but the high proportion of FL in the composite lymphoma favors its presence at least in that component. The factors that enable EBV-driven lymphoma transformation remain to be further elucidated, though data are emerging to this end [5].

From a diagnostic standpoint, EBV + DLBCL can present as a monomorphic form, a polymorphic form and, occasionally, as a gray zone between DLBCL and cHL [12]. According to the World Health Organization and International Consensus Classification criteria, the latter cases should be diagnosed as EBV + DLBCL [13, 14]. Appropriate therapy for all morphologic types of EBV + DLBCL is generally considered to be like that for EBV- DLBCL. However, this case showed a distinct component with cHL morphology and immunophenotype, albeit representing a minor portion of the biopsied tissue. Moreover, persistence of only EBV + cHL following multiple lines of B-cell directed therapy (R-CHOP, the trial regimen suspected to be loncastuximab plus rituximab due to total CD19 negativity of relapse biopsy) further supported the distinct biology of the cHL component.

This case highlights the importance of careful assessment of EBV + DLBCL, especially the polymorphic form, to exclude cHL due to different therapeutic implications. Lymphoma cell morphology, background cellularity, nodal versus extranodal presentation, and comprehensive immunophenotypic assessment of the B-cell program (CD20, PAX5, OCT2, BOB1, and CD79a) are all critical for the differential diagnosis between polymorphic EBV + DLBCL and EBV + cHL [13]. Thorough diagnostic evaluation often requires excisional biopsy and occasionally necessitates image guidance with PET/CT to identify FDG-avid sites of involvement. Failure to exhaustively assess all components of a surgical specimen may result in ineffective therapy and selective outgrowth of aggressive components of a lymphoma. Furthermore, the eradication of DLBCL and FL components but persistence and subsequent overt relapse as cHL in this case raises the question as to whether the identification of composite lymphomas with both DLBCL and cHL should alter initial therapy [15]. An ongoing clinical trial of brentuximab vedotin in EBV + DLBCL may support future initial therapy with activity against both lymphomas (https://clinicaltrials.gov/ct2/show/NCT01671813).

## Figures and Tables

**Table 1:** B-cell receptor clonality study peak sizes of distinct composite lymphoma components.

Tissue source	FR1^*∗*^	FR2	FR3	KA	KB
Follicular lymphoma	X	X	X	293.4	277.59; 281.67
Initial EBV + DLBCL	329.16	264.11	X	293.36	277.47; 281.69
Relapsed EBV + cHL	328.99	263.89	X	293.37	277.48; 281.69

^*∗*^PCR fragment size (nt); FR: Ig heavy chain framework; KA, KB: Ig kappa light chain A, B primer sets.

## Data Availability

The clinical and pathologic data used to support the findings of this study are included within the article.
